# Supplementary material for: Prescription of concomitant medications in patients treated with Nifurtimox Eflornithine Combination Therapy (NECT) for T.b. gambiense second stage sleeping sickness in the Democratic Republic of the Congo
Source: PLoS Negl Trop Dis. 2020 Jan 27;14(1):e0008028. doi: 10.1371/journal.pntd.0008028 (PMC7004379; doi:10.1371/journal.pntd.0008028)
Supplement: Attachment — Submitted filename: NECT-FIELD comed_PLoS NTD_Response reviewers_20191211.docx [file pntd.0008028.s001.docx]

The reviewers’ comments are left in italics and our responses are inserted in the text in normal font.

**Responses to reviewer #1**

**General comments**

*Methods are very simple, just a data summary, all from one clinical trial. But in order to support some of their conclusions, they could add some data from routine practice outside clinical trials, some data on cost, etc, to allow comparisons that they insinuate in a superficial way.*

*Results are clearly presented, although in a minimalist way. From that clinical trial, the results of most relevance for public health (the effectiveness of NECT), are not presented.*

*Several conclusions are not supported by the data presented.*

Response from authors: We acknowledge that we opted for a very simple method and that we presented results in a factual way. According to our latest literature research, concomitant medications used in the frame of sleeping sickness were never published so far. With this publication we aimed at filling this gap. In order to bring more evidence, we plan to present the concomitant medication used with fexinidazole and acoziborole once all data will be collected and analyzed. We addressed this limitation in the discussion.

The 24-months follow-up effectiveness results of the NECT-FIELD study were presented to the scientific community at the 32rd ISCTRC conference in Khartoum in September 2013. We plan to submit the publication relating these results early 2020 to PLoS NTD. Regarding the chronology of publication, we preferred prioritizing this present publication as it is based on in-hospital co-medication prescription and therefore directly linked to the first publication by Schmid et al (2012).

We acknowledged your comment advising us to add routine practice data outside clinical trial and data on cost. Unfortunately, adding these additional data would mean additional data collection work that is not feasible within the scope of this present work. However, as comparability and generalizability is a limitation of this study, we added a summary table of the most frequent adverse event that occurred in other clinical trials and field studies using DFMO, NECT and fexinidazole in order to be able to compare different settings and therapies and we also discussed this point in the discussion.

Finally, the discussion and conclusions of this paper were co-written with and approved by all NECT-FIELD investigators and members of the Congolese HAT program who are cited in the authorship. They gave us their perspective about best treatment options and clinical care currently available according to current clinical practice and guidelines. We adapted the discussion and conclusion accordingly.

**Editorial and Data Presentation Modifications**

*No comments*

**Summary and General Comments**

*This paper provides a description of the concomitant medication given to patients included in a trial, known as NECT-FIELD, evaluating the NECT treatment for second stage sleeping sickness.*

*The first and principal comment is that the most important results of that trial, the efficacy of NECT in the real-life setting, in children, in pregnant and lactating women, were not published, and are not available anywhere. These data are much more awaited by the scientific and public health community, than these data on concomitant medication.*

*The trial was successfully completed several years ago, including the post-treatment follow-up, and the effectiveness data should be published, taking priority to the data presented in this paper.*

*Now regarding this article, the results presented are simply an enumeration of medicines given and their frequency. Only in the discussion the authors make some critique and suggest possible improvements, but without explaining their critique nor providing any data supporting the changes proposed.*

*Line 77: The EMA’s opinion did not recommend fexinidazole for all second stage patients. They excluded “stage 2 patients with >100 WBC per μL of CSF”. The authors should use the appropriate references for this, such as the EMA report available in their website, or the article published by EMA authors in PLOS NTD: Pelfrene et al. The European Medicines Agency's scientific opinion on oral fexinidazole for human African trypanosomiasis. PLoS Negl Trop Dis. 2019 Jun 27;13(6):e0007381*

Response from authors: We agree with the suggestion, modified the manuscript accordingly, and updated the reference with Pelfrene et al and new WHO interim guidelines.

*Lines 80-83: For a complete presentation of the NECT-FIELD study, the authors should not forget to mention that efficacy was also documented, but never published.*

Response from authors: We adapted the sentence in order to avoid any confusion about the effectiveness results that will be submitted for publication early 2020.

*Line 96: Instead of “design, methods, and results”, it should be “design, methods, and safety results” (because efficacy results were not included in the Schmid paper).*

Response from authors: We agree with the suggestion and modified the manuscript accordingly.

*Line 103: As these patients were all enrolled in a clinical trial, is it true that “Concomitant medication was prescribed according to the decision of the investigator and the local guidelines.”? Didn’t the study protocol impose any concomitant medication? This is important in the interpretation of results. How much these results represent the practice in DRC (or elsewhere) outside clinical trials?*

Response from authors: Investigators did not receive any guidelines on how to prescribe concomitant medications. Except receiving a medical training at start and having an emergency pharmacy for treating life-threatening issues on site (which we considered as an ethical obligation), they were not influenced in their prescription practice. The objective of the NECT-FIELD study was to have a formal understanding of how NECT was used in real life conditions while NECT was being deployed in the field. We added a sentence for clarification: “An emergency pharmacy for eventual life-threatening issues was provided by the study coordination to the Investigators while the other drugs were routinely supplied by the national HAT program or the hospital pharmacy”.

*Lines 181-188: It’s not clear why the authors focus on the encephalopathic syndrome linked to melarsoprol, and they seem to judge the appropriateness of use of certain drugs related to this syndrome, which does not even appear among the AEs in the study (and none of the patients received melarsoprol). It would be logical that they assess the use of drugs in relation to the AEs observed.*

Response from authors: We agree that the comparison with the encephalopathic syndrome described with melarsoprol is confusing and not evidence-based. We adapted the paragraph accordingly and only focus on the treatment of neurological AEs without drawing any conclusions about encephalopathic syndrome.

*Lines 193-195: The authors say that using metamizole and papaverine is inadequate. They don’t explain why, except that they are “banned in many countries with stringent drug regulation”. This argumentation is way too superficial to be acceptable. Just by searching a little I can see that metamizole is banned only in a few countries (UK, Sweden, USA, Canada) because of agranulocytosis observed in people from northern Europe, or having population from that origin. Other countries use it widely and don’t have the same problem. India banned it at some point but they lifted the ban. The only argument offered by the authors would then be that metamizole should not be used in Africa because it provokes problems in northern Europeans (!). But perhaps the authors have other data that they can share, to support their recommendations. There are at least 2 recent review papers on metamizole published, which have much different conclusions: one says “For short-term use in the hospital setting, metamizole seems to be a safe choice when compared to other widely used analgesics.” I didn’t spend time searching on papaverine, but the authors should.*

Response from authors: Thank you for this important comment. We discussed it with all co-authors and modified our interpretation of the results as follows: “It also appeared that some classical field prescription practices such as the utilisation of metamizol and papaverine, administered in 25.4% and 2.5% of the patients respectively might be considered as suboptimal at first sight. However, after discussion with the investigators these choices were either driven by the availability of drugs at the centres or by some medical decisions. At the time of the study, between 2008 and 2010, metamizol, due to the suspicion of bone marrow toxicity, was only recommended when other antipyretics were inefficient or when the fever needed to be brought down urgently, above all in children. In this specific population, metamizol was often preferred because the intravenous route made its administration easier. Neverthelesss, metamizol has been banned in DRC since and treatment with NSAID such as ibuprofen or diclofenac would therefore be recommended today. Papaverine was administered preferably to pregnant women to treat gastro intestinal disorders such as nausea and vomiting, because metoclopramide is contra-indicated in this population”.

*Line 195: Perhaps the statement “inadequate choices in the drug supply of the centres were mainly driven by the scarce financial resources available”, could be supported with some concrete examples giving the cost of choices made, versus the cost of the alternatives.*

Response from authors: this issue was discussed with the co-authors who conducted the field work and we all agreed to adapt the sentence as follows: “In a low-resource setting, drug supply choices of the centers may partially be driven by the scarce financial resources but also by their availability at the hospital and surrounding pharmacies or whether the drug was supplied by the national HAT program. For example, the choice to prescribe levamisole and cotrimoxazole was mainly driven by the fact that these drugs were supplied by the HAT national program and their availability. However, data on concomitant drug supply and availability have not been collected at the time of the NECT-FIELD study. Therefore we cannot conclude on the relative importance of how lack of resources, supply chain, availability, and access influenced the utilization of concomitant drugs in our setting.”

**Responses to reviewer #2**

**General comments**

*The objectives of the study are well stated. The study design is appropriate for the stated objectives. Study population has been well described in the methodology and the sample size is sufficient to power this study. Descriptive statistics were used and this suffices for the kind of study objectives that the authors set out to determine. Being review of secondary data from a clinical trial, ethical concerns were adequately addressed.*

*The results presented match the analysis plan. Results were presented in an exhaustive and logical manner, from baseline characteristics to the main findings in line with the objectives.The tables are clear and well structured (columns and rows are relevant).*

*The authors have described the most common types of concomitant medications prescribed in patients with late stage sleeping sickness. These include analgesics/antipyretics, antimalarial and anti-helminthic drugs. They further go ahead to suggest a list of medicines that should be availed alongside NECT.*

*The resource-limited setting of occurrence and treatment of sleeping sickness implies that preparations for adverse event management is defective. Various treatment centres are likely to have varying stock levels for the concomitant medicines that they may need at a certain point. The suggestions from authors contributes to the standardization of the medicines list.*

*These claims are novel and demonstrates the authors’ deep understanding of the gaps and therefore the needs at hand.*

*The context of the findings is also in line with the rest of the other sections of the manuscript.*

*The paper is outstanding in the field of treatment for sleeping sickness. The disease presentation in itself often warrants concomitant medicines because of the severity of some presenting symptoms. Similarly, some adverse events experienced during the course of treatment can be life threatening. The authors were able to differentiate these two time points. This gives insight into the requirements before and during treatment.*

Response from authors: No issue was raised from the comments.

**Editorial and Data Presentation Modifications**

*Lines 101 and 102 reads: ‘Prior to NECT, the patients received standard pre-treatment according to the national guidelines, consisting of antimalarial, anthelminthic, and, if required, antipyretic/analgesic medication.’ This gives an impression that all patients receive antimalarial as a pre-requisite. Please confirm this.*

*This contradicts line 37/38 where anti-malarial medicine is classified as ‘additional’.*

Response from authors: Yes according to national treatment guidelines in DRC, all patients were “de-parasitized” before start of NECT. This comprised antimalarial and anthelminthic drugs. Antipyretic and analgesic drugs were added if necessary on a symptomatology basis.

We understand the incoherence with the words “additional treatment” that we replaced by “concomitant treatments medications administered during the hospitalization period before and during NECT” (line 37/38). Later in the text, we replaced “additional treatment” with “concomitant treatment”.

*Line 40 and 41 reads ‘Treatment of encephalopathic syndrome or similar neurological disorders was used in a relevant proportion of patients (approximately 5%)’. The word ‘relevant’ seems out of place. Did you mean ‘significant’?*

Response from authors: We agree with your suggestion and modified the manuscript.

*Line 44 reads ‘…even if some prescription habits would need to be adapted in the future’. it is not well aligned to the statement preceding it. Gives a different meaning.*

Response from authors: We understand that there is a syntax issue. We modified the sentence into: “The prescription of concomitant treatment was coherent with the clinical and safety profile of the patients. However, some prescription habits would need to be adapted in the future to the evolution of the available pharmacopoeia.”

*The title should specify that participants had T.b gambiense.*

Response from authors: We agree and modified the title.

**Summary and General Comments**

In summary, there are no copyright issues to be concerned about. The manuscript has been submitted for consideration in line with earlier work done by the same authors.

Additionally, ethical principles have been followed as by virtue of the details in the section with the same title and the methodology.

Clarifications based on the comments in the above.

Response from authors: No issue was raised from the comments.

**Responses to reviewer # 3**

**General comments**

*The objective of the study are clear: indication of the use of concomitant medication during HAT treatment. The study design is appropriate and the population clearly described. The sample size is adequate and correct statistical analysis provided. Ethical committees in both countries have been consulted.*

*The analysis is correct and the results clear. The tables are complex but complete.*

*The conclusions are clear and the limitations shortly described. The results should help to implement concomitant medication for the treatment of HAT.*

Response from authors: No issue was raised from the comments.

**Editorial and Data Presentation Modifications**

*Line 96: should be "the NECT-FIELD study" instead of NECT-FIELD.*

Response from authors: We agree with the suggestion and modified the manuscript.

*Line 112: not really clear the sentence "AEs in more than 5% of the patients and concomitant medications in more than 2.5%"? Could it be clarified?*

Response from authors: We understand that the sentence is too vague for the reader. We changed the sentence into: AEs observed in ≥ 5% of the patients and concomitant medications prescribed in ≥ 2.5% have been included in the result tables for the assessment of concomitant medication prescriptions.

*Line 136 and table 2 : "such as neurological, sleeping and..." the sentence neurological does not mean anything here : has to be removed also I do not understand why there are no other neurological signs described at least abnormal movements and walking disorders which are characteristic of advanced stage.*

Response from authors: We removed the sentence. Due to the field context, we simplified as much as possible the data collection and the case report form (CRF) in order to diminish the burden of investigator’s work as much as possible. On admission, the investigators had a list of signs and symptoms to check if the patient was complaining about (this is reported in table 2). Unfortunately abnormal movement was not retained and put in this list. Nevertheless, walking disorders are present (see table 2).

*Table 3: not clear what SOC and LLT means and makes in the first line of the table?*

Response from authors: SOC and LLT comes from the MedDRA (Medical Dictionary for Regulatory Activities). MedDRA is organized in five levels of coding, like a hierarchy. The highest level is the system organ class (SOC), e.g. Nervous system disorder. The lowest level is the Lowest Level Term (LLT), e.g. headache (the adverse event). We deleted these acronyms, as they are not necessary to understand the table.

**Summary and General Comments**

The paper provides an opportunity to clarify what kind of concomitant medication should be available in HAT treating centers. As the number of HAT cases is low, all additional data from well conducted trials are of great interest for the general use and are thus interesting to show to the scientific community and policy makers

Response from authors: No issue was raised from the comments.
